# The Management of Perinatal Borderline Personality Disorder

**DOI:** 10.3390/jcm12216850

**Published:** 2023-10-30

**Authors:** Anne Sved Williams, Rebecca Hill

**Affiliations:** 1 Women’s and Children’s Health Network, North Adelaide 5006, Australia; rebecca.hill@sa.gov.au; 2 Faculty of Psychiatry, University of Adelaide, Adelaide 5005, Australia; 3 School of Medicine and Psychology, Australian National University, Canberra 2601, Australia

**Keywords:** borderline personality disorder, perinatal, management, infant, dialectical behaviour therapy, mother-baby unit

## Abstract

Borderline personality disorder (BPD) is highly prevalent in clinical perinatal mental health settings, although there are few systematic programmes to identify BPD at this time. Retrospective studies show compromised birth outcomes for women with this condition, and several authors have highlighted a significant range of problem outcomes for offspring identifiable from early infancy through the adult years, including the intergenerational transfer of mental health problems from mother to child. A literature review identifies the varying prevalence rates found in non-clinical and clinical settings and the paucity of published studies on the management of perinatal BPD, in particular focused both on the mother and mother-infant relationships. A case study is presented to show both the potential benefits of inpatient mother-baby unit protocols and of specialised longer-term group therapy. Many knowledge gaps can be identified for further clinical research that could potentially benefit families with perinatal BPD, including systematic identification of perinatal BPD and intensive programmes that not only could perhaps improve birth outcomes but also provide skills to mothers to help with their emotional regulation and potentially improve mother-infant relationships and longer-term offspring developmental pathways.

## 1. Introduction

Borderline personality disorder (BPD), as defined by Diagnostic and Statistical Manual 5 [1], is diagnosed by the presence of five or more of nine criteria, particularly centred around emotional dysregulation (ED). Not surprisingly, pregnancy, childbirth, and parenting are times potentially fraught for women with this personality style, with many factors contributing. These include body changes in pregnancy and the interactions with health professionals in pregnancy and birthing [2] for those many women for whom childhood sexual abuse was an antecedent of their personality-related issues. Intrapartum birth trauma is increasingly identified as problematic [3]. Postnatally, multiple impacts include sleep deprivation, crying infants [4], and changing family dynamics. Given a childhood often formed by troubled relationships with their own parents, many also lack a sense of parenting competence [5]. A recent systematic review of the prevalence of pBPD [6] confirms that many women present perinatally for care. Their work could be considered to inadvertently focus concerns on the paucity of management options, which were identified in another systematic review [7]. This overview will therefore review perinatal BPD, its effects on infants, and care and treatment options for women with pBPD, highlighted by a case presentation that shares local practices. Many gaps in research have been identified.

## 2. Prevalence

Estimates of community prevalence of BPD vary greatly from 1 to 6% [8,9], and estimates of perinatal BPD also vary widely, with the paucity of studies on the prevalence of this condition making definitive findings difficult. Howard et al. [10] found 0.7% of antenatal women with a BPD diagnosis on SCID interviews, but in an internet-based Canadian study [11] of 590 women screened in an antenatal setting with a range of mental health self-report questionnaires, 12.5% of women identified the presence of BPD on the McLean Screening Instrument for BPD (MSI-BPD), a substantial cohort. Prasad et al. [6] highlighted up to 35% of women with pBPD in clinical (mental health) presentations. There were much higher rates in some settings, e.g., mother-baby units, with an overall pooled prevalence rate of 14% in available studies of this condition in perinatal women, mostly postnatal. However, they were only able to identify seven studies in total to include in their meta-analysis. This contrasts starkly with perinatal depression. A recent publication found 128 META analyses of PND [12]. It is clear that the small number of studies of perinatal BPD in only a few countries needs replication more widely and systematically.

### 2.1. BPD Effects on Offspring

There has been little research on systematic antenatal identification of BPD or prospectively documenting the effects of this condition on birth outcomes. Judd et al. [13] note that screening with the Edinburgh Postnatal Depression Scale will not infrequently identify BPD. Birth outcomes in clinical populations in retrospective studies were found to be compromised, including premature delivery, low APGARs, and increased use of special care nurseries [14]. In addition to these findings, Pare-Miron et al. [15] described premature rupture of membranes, higher rates of gestational diabetes, a younger mother, and higher rates of Caesarean section.

Postnatally, the mental health difficulties experienced by the women may significantly impact their ability to parent, with consequences for their children. Several authors [16,17,18] have summarised the effects of pBPD on offspring. It is clear that compromised interactions can be identified early in life. For instance, Kiel et al. [19] found that, compared to mothers with low BPD symptoms, mothers with high BPD symptoms stay positive with their distressed infants for shorter periods of time and are increasingly less sensitive as their infants continue to show their distress. A later study by the same group [20] suggests that maternal emotional dysregulation may be of greatest significance in infant emotion regulation. A recent study [21] analysed the interactions of 25 women with BPD and 29 women without this condition as they interacted with their toddlers aged 18–36 months. They found that for dyads with a BPD mother, there was less synchrony between mother and infant, perhaps because these mothers, despite responding to their infants distress equally to the mothers without this condition, may have been less effective in achieving that synchrony.

Not surprisingly, problems are identified in the older children of mothers with pBPD. Prospective studies of the work of Stepp and others summarised well by Petfield et al. [16] and Eyden et al. [18] show internalising and externalising symptoms in childhood, followed by a range of mental health symptoms in adolescence and the intergenerational transfer of BPD in young adult life [22,23,24].

It is also apparent that mothers with BPD are frequently aware that their models for parenting could be flawed and that without new skills and knowledge, intergenerational problems in their infants are possible [25]. Zalewski et al. [26] also explored similar themes and invited participants to identify what therapeutic interventions may be helpful. Themes the 23 mothers identified included their wish that parenting information was integrated into their BPD skills groups and how that might usefully happen. There were strong themes of stress, uncertainty, and guilt they felt as parents, with an awareness of deficits from their own upbringing.

### 2.2. Diagnostic Identification

Careful clinical evaluation will allow the clinician to tease out the features of BPD described in DSMV as well as co-morbidities if present. In addition, it is vital to understand how postnatal women are managing their infant(s) and other family relationships and provide relevant management.

Misdiagnosis or failure to provide psychoeducation because of stigmatisation has been the norm until recently for people with BPD [27]. Internet use has led to significant self-diagnosis [28], although there is still a general preference towards clinician diagnosis [28]. Additionally, in general (non-perinatal) clinical psychiatric populations, there is substantial overlap with other psychiatric disorders with emotional dysregulation [29], particularly cPTSD [30], and also bipolar mood disorder [31]. Adult attention deficit hyperactivity disorder (ADHD) is another diagnosis in contention for many people with emotional dysregulation [32]. Studies of the overlap of these conditions in perinatal populations could not be identified. Diagnostic clarification is vital for appropriate treatment, but challenging.

Co-morbidities with all these conditions are common [33], as are substance use [34] and depression [35]. Not surprisingly, those studies that have looked for the comorbidity of BPD in perinatal women have found significant overlaps, both antenatally [13] and postnatally [34]. Co-morbidity may be particularly common in mother-baby units, where many women are admitted with a diagnosis of postnatal depression [36]. The identification of high rates of BPD in admitted mothers in our centre [37] led to a change of practice. In that study, 23% had a confirmed clinical diagnosis of BPD, while 46% were found to identify with those features on self-report questionnaires. Anecdotally, we have found that when the nine criteria of BPD in DSM are explored collaboratively with affected mothers and the diagnosis is definitively made, almost all women voice very positive views of the provision of a diagnosis of BPD. A diagnosis with which they can identify provides a pathway to understand their situation and to move towards a treatment plan.

### 2.3. Management

Despite the prevalence as described and the known effects of maternal BPD on birth outcomes and infants, the literature on clinical management for this troubled group of women and their infants is sparse. Indeed, again, the comparison with the treatment of postnatal depression (PND) is stark. While the prevalence of these conditions in clinical perinatal populations now appears to be similar at about 14% [6,38], the literature reviews of the management of PND reveal many studies on a large range of treatments that will help guide management choices. These include extensive controlled trials of psychotherapy [39], medication [40], and a range of other treatments, e.g., omega 3 [41], and exercise [42]. In contrast, a recent systematic review of interventions for pBPD [7] found just 7 studies, mostly group treatments using variations of dialectical behaviour therapy [43], overall providing little guidance to clinicians in how to manage the condition. A search for medication management for pBPD found only case studies, e.g., Gentile [44]. A Cochrane review [45] confirmed previous reviews that medication has extremely limited value in general BPD populations. Combined with women’s reluctance to take psychotropic medications during pregnancy and lactation [46], it is unlikely that future reviews of pBPD management will find many studies of medication for perinatal BPD.

Alongside the potential beneficial management pathways provided in the current literature, a clinical case scenario is provided in two parts (inpatient and community management) to highlight the challenges that arise in the treatment of affected dyads and local protocols that may be useful.

## 3. Inpatient Management in a Parent-Infant Inpatient Unit

While the prevalence of pBPD in mother-baby units (MBUs) is known to be substantial and improvement at discharge is known to be compromised [36,37], few authors have explored inpatient management. Bittner et al. [47] found higher psychopathology and more problems in mother-infant relationships at discharge in mothers with maladaptive personality styles compared to mothers without such styles, although their focus was not only on pBPD. Howard et al. [48] noted that the infants of mothers with schizophrenia or BPD were more likely to require social services supervision after discharge from an MBU compared to mothers with other diagnoses. The authors of these and similar articles conclude that inpatient management could well include a management plan specifically tailored for this population but do not describe specific treatment.

Because of the high rates identified in our unit [37], the clinical team developed a protocol for the inpatient management of BPD. Steps include early identification of this diagnosis, which is frequently comorbid with depression in women whose referrers generally describe the woman as having PND. If BPD is present and identified either on intake interviews or becoming evident during the early days of admission, after team discussions and agreement, a senior team member works through the criteria of BPD from DSMV, with clinician and patient moving towards agreement if appropriate.

This interview is a therapeutic intervention in its own right. There is an emphasis on making links between her early experience of invalidating or abusive environments where identified and the symptoms from which she suffers, many of which can be seen to be a product of the reactions or skills that she needed to survive. Thus, the interview serves multiple purposes: diagnosis, psychoeducation, meaning-making, stigma-busting, and the instillation of hope by advising her that others suffer in similar ways and that research has been devoted to the development of effective treatments. Links to the difficulties in parenting are highlighted, and this is often a time for women to express their fear that they will not parent their offspring well and their desire to do better than their parents.

At this time, the ward’s BPD protocol is discussed, with patients provided a maximum of fourteen further days in the unit, thus ensuring a specific discharge date is discussed early on. The intense relational experiences in the unit, when combined with core symptoms of BPD such as abandonment sensitivity, emotional dysregulation, identity diffusion, and fears around parenting, can make discharge a painful and disorganised prospect, which we find is usually contained sufficiently by the simple manoeuvre of setting a known discharge date. In this unit, the average length of stay is about three weeks, and most women with pBPD will have a similar length of stay since the institution of this protocol. Previously, escalation of suicidal ideation in the days prior to discharge was common, often leading to prolonged admissions without great additional therapeutic benefit.

Where possible, a primary therapist is allocated, and the woman is provided with psychoeducation about pBPD, generally with very positive acceptance. Information is given in verbal, written and website-based formats. During the next fourteen days, the woman receives teaching on mindfulness and distress tolerance, as well as routine MBU care and a focus on helping her with infant settling. It is clear that for many women with BPD, infant crying is a trigger for maternal distress and withdrawal, leaving the infant potentially without care as well as heightening the woman’s fear of her capacity to parent [4]. Clinicians aim to support the woman in successfully managing her emotions while staying present with her infant.

A key part of the implementation of this protocol has been a long-running programme of education about BPD for all levels of clinical staff, so that all members of the team are able to coach and encourage patients in their understanding and acceptance of the diagnosis, along with new skills to manage their own and their infant’s distress.

### Cynthia and Henry: Clinical Case Part 1: Inpatient Management

Cynthia * was 29 when she delivered her first infant, a son, Henry * (* Cynthia and Henry are fictitious names). She reported that motherhood had started disastrously, with the midwife attending her and leaving her 10 min prior to delivery as her shift had finished. Prone to abandonment sensitivity, when her infant was born, Cynthia was already distressed, so when Henry was immediately taken to a special care nursery with urgent health issues, her sense of herself as a mother was challenged. She reacted with increasing emotional turbulence when the policies of the nursery precluded night visits, and her parenting competence was further challenged when breast feeding failed. An additional concern was her lack of connection to Henry.

She soon became profoundly depressed and actively suicidal, and she was admitted to our mother-baby unit when Henry was five weeks old. ECT was commenced as a preferred treatment for her diagnosed major depression. As her mood gradually improved and there was further review of her history, several facets of her story led clinicians to discuss with her the nine criteria of BPD, of which she identified seven as being strongly in her life. These included self-harm by cutting as a teenager, emotional dysregulation and angry outbursts, extreme sensitivity to rejection, a range of highly ambivalent friendships, and impulsive behaviours including binge drinking and fast driving. She received a BPD diagnosis that had not been provided to her prior to her pregnancy in previous therapies with several psychologists for symptoms of depression and anxiety. Either the full range of symptoms had not been explored or were not as evident until the needs of her infant triggered an escalation. Dual diagnoses of major depression and BPD were considered appropriate by both patients and staff. The protocol for BPD described above commenced towards the end of the ECT course. Henry was notably gaze-avoidant with Cynthia, who confessed to feeling that she did not like him. These aspects responded positively to a series of mother-infant therapy sessions employing video reviews of their interactions.

Cynthia was discharged within two weeks of the protocol institution, with referrals to a range of community supports. As is typical, however, due to resourcing issues, these services were not immediately available at the point of discharge. In particular, many women wait for many months for specialised services such as mother-infant dialectical behaviour therapy (MI-DBT) (Sved Williams et al., 2021 [49]). Thus, soon after discharge, Cynthia suffered a further deterioration in mood and an escalation of self-harm. This led to admission to an adult mental health facility, where a diagnosis of bipolar mood disorder was made and mood stabilisers were commenced, with a range of side effects that were poorly tolerated, leading to further deterioration and a suicide attempt by overdose in the inpatient unit, requiring urgent treatment. At discharge, after liaison with our team, the community plan made in the MBU was reinstated, with resources now available, and Cynthia was discharged home and reunited with her infant and highly supportive partner. Her community treatment is discussed later.

## 4. Community Management of Perinatal Borderline Personality Disorder

Recent reviews of the treatment of BPD [50,51,52] have provided optimism regarding the programme benefits of psychological interventions tailored for BPD. Several treatments, including schema therapy, mentalisation-based therapy (MBT), and dialectical behaviour therapy (DBT), show evidence of positive therapeutic effects by group conclusion, which can be sustained over time. Structured therapies, including MBT and DBT, may provide greater benefit than more general psychotherapy for specific BPD symptomatologies such as suicidal ideation and behaviours [53].

For mothers with BPD, the complexity of parenting is often a new burden [5]. Zalewski et al. [26] explored the concerns of women with pBPD. Women completing this study clearly defined their wish that information on parenting should be integrated with or added to therapies such as DBT. This study made it clear that standard treatments for BPD were less useful to them.

However, despite the evidence of the efficacy of some BPD therapies and mothers’ stated wishes, given the recency of evidence-based structured therapies for people with BPD, it is perhaps no surprise that there are few publications on the community treatment of pBPD, although some recently developed programmes may well serve a similar population where maternal trauma is central to inclusion [54,55].

The few earlier studies of perinatal BPD treatment tend to be case-based. Wendland and colleagues [56] provide a skilled account of the psychodynamic world that women with BPD may inhabit and the complexity of treatment. This article is a very good review of the influencing factors in one woman’s presentation and the parenting issues raised. The woman described has very severe problems; there are clear ongoing child protection concerns, and the therapy offered is extensive both in terms of the time over which the woman and her infant were seen (three years) and also the numbers of practitioners involved, with individual, dyadic co-therapy and group therapy offered at various times. This case illustrates that even with such long-term commitment, the childhood trauma suffered by the woman has left problems that require ongoing therapy, even with a motivated patient and a well-resourced and trained team. Practical considerations of access, both in terms of therapist time and cost, are likely to make such extensive treatment unavailable to most families.

Other approaches, mainly using group work, have been trialled. In their review, Florange and Herpertz [17] focus on specialised therapies that have been adapted for perinatal women. They identify three. Renneberg and Rosenbach [57] describe practitioner and client views of a new programme, Parenting Skills for mothers with BPD, which showed promise, although outcome measures related to the acceptability of the programme rather than its clinical utility. Recently, these authors and others [58] have outlined the study protocol for a controlled trial of this 12-session programme, which will focus on outcomes for mothers with BPD who have an intervention compared to a waiting list control. A second programme described by Florange and Herpertz [17], Coming up for Air, is a four-session intervention that provides information on parenting and BPD, but thus far the information provided relates only to clinician acceptability and not to clinical outcomes [59].

A third programme, developed at our centre [49], is a 24-session adaptation of group DBT that intersperses parenting and attachment information with the learning of DBT skills—mother-infant DBT (MI-DBT). While evaluations show improvement in all measures of maternal mental health, including BPD symptoms, depression, and anxiety, there is less clarity about impacts on infant development, and there are no control groups. A recent adaptation of MI-DBT currently under way (MI-DBT+) provides an additional 10 weekly sessions of an evidence-based mother-infant therapy, Attachment and Biobehavioral Catchup (ABC) [60]. ABC has provided benefits to many cohorts but has not previously been trialled for women with pBPD. Results for MI-DBT+ are not yet available.

Retention rates for MI-DBT groups are 70%. A recent article [61] reveals the extent of dropouts from group therapy in non-perinatal situations at 48%, although Iliakis et al. [62] found a 70% completion rate with individuals, comparable to MI-DBT groups. A recent qualitative study highlights the women’s positive views of MI-DBT [25] and their motivation to be good mothers, which is likely to enhance attendance/retention rates, as is the on-site provision of childcare.

Documented long-term outcomes for BPD also provide some hope that interventions work. Gillespie et al. [63] find that at one year post-therapy, it is likely that positive changes remain for those undertaking DBT. Temes and Zanarini [64] report that remission is the norm for the majority of patients with BPD and that full recovery occurs over time, although a similar study [65] concerningly notes that women have lower functional improvement. They offer no explanation for this finding, although in most countries around the world it is clear that women continue to bear the work of caregiving, in particular parenting and other relational activities such as elder care, which involve repeated exposure to difficult emotions. For women with pBPD, interrupting the potentially compromised relationship between a troubled mother and her child, who may well be developing problems in their own right, may be hypothesised as a key to changing these trajectories for women, but no studies are available to clarify this hypothesis.

### Cynthia and Henry: Clinical Case Part 2: Community Management

Cynthia was able to commence MI-DBT after her discharge from the adult inpatient service, and by this time, her community care was undertaken by a community psychiatrist who provided a diagnosis of cPTSD, which Cynthia preferred to either BPAD or BPD, and she ceased the mood stabilisers. Cynthia rapidly learned and implemented the skills of MI-DBT, and her emotional ability improved substantially. She then began to attend and complete a course of adult learning. Prior to therapy, her frequent episodes of emotional lability had interfered with course completion, and she had been largely unemployed. She qualified as a technician in a much-sought-after field, therefore rapidly finding work. When interviewed two years after finishing MIDBT, she described stability in her life never previously present, including changes in the marital relationship she had been easily able to negotiate with her very supportive partner, who had sought out psychoeducation about BPD. She reported a very loving relationship now with her child, Henry, who was developing well. She attributed the improvement to learning about parenting during MI-DBT. She and her partner were content with their one child, unprepared for the potential disruption that a second child might bring, despite Cynthia’s clarity regarding her parenting competence.

## 5. Conclusions

Women with pBPD want and deserve treatment for themselves from evidence-based programmes, and their infants also need early interventions to potentially optimise developmental pathways. Many women are motivated by parenthood to address long-standing behaviours and mental health concerns. Despite evidence of the frequency of BPD, including pBPD, and the effects on their infants, very few programmes offer care for the woman in her own right and for herself as a parent, despite growing evidence of the efficacy of such interventions. There is still insufficient evidence that therapeutic interventions for the mother will change potentially problematic life trajectories for their infants; however, the plausibility of this hypothesis is compelling and warrants urgent investigation.

Many questions remain in this highly under-researched area. Antenatal prevalence of BPD has not been systematically studied by routine identification, and no prospective longitudinal studies have followed pregnant women to document whether incidence rises postnatally. No intervention studies could be accessed that provide information on the potential for improving birth outcomes and the early postnatal course in women who do identify with BPD criteria in pregnancy.

The significant number of women presenting postnatally for care compared to the numbers of women identified with BPD in antenatal clinics [10] suggests that either birth itself, hormonal changes of parturition and breast-feeding, infant care, or a combination of these could be psychologically overwhelming for women who are prone to emotional dysregulation. Would providing knowledge and skills to these mothers either antenatally or in the first weeks of their infants’ lives set different trajectories?

Partner relationships and their place in the lives of women with pBPD is a topic that has also received little attention. How much do partners contribute to or help prevent exacerbations of emotional dysregulation, and in what situations would inclusion of partners show particular benefit? What is the place of comorbid diagnoses such as depression, cPTSD, and ADHD? Treatments for pBPD, such as MI-DBT, help women with cPTSD, as demonstrated by the overlap of symptoms in Cynthia’s presentations. The rewards of interventions can be lifelong for both mothers and particularly infants, and with better acceptance of the reality of the health and developmental effects of mental health problems, perhaps research will find answers over time.

## Data Availability

No new data were created or analyzed in this study. Data sharing is not applicable to this article.
